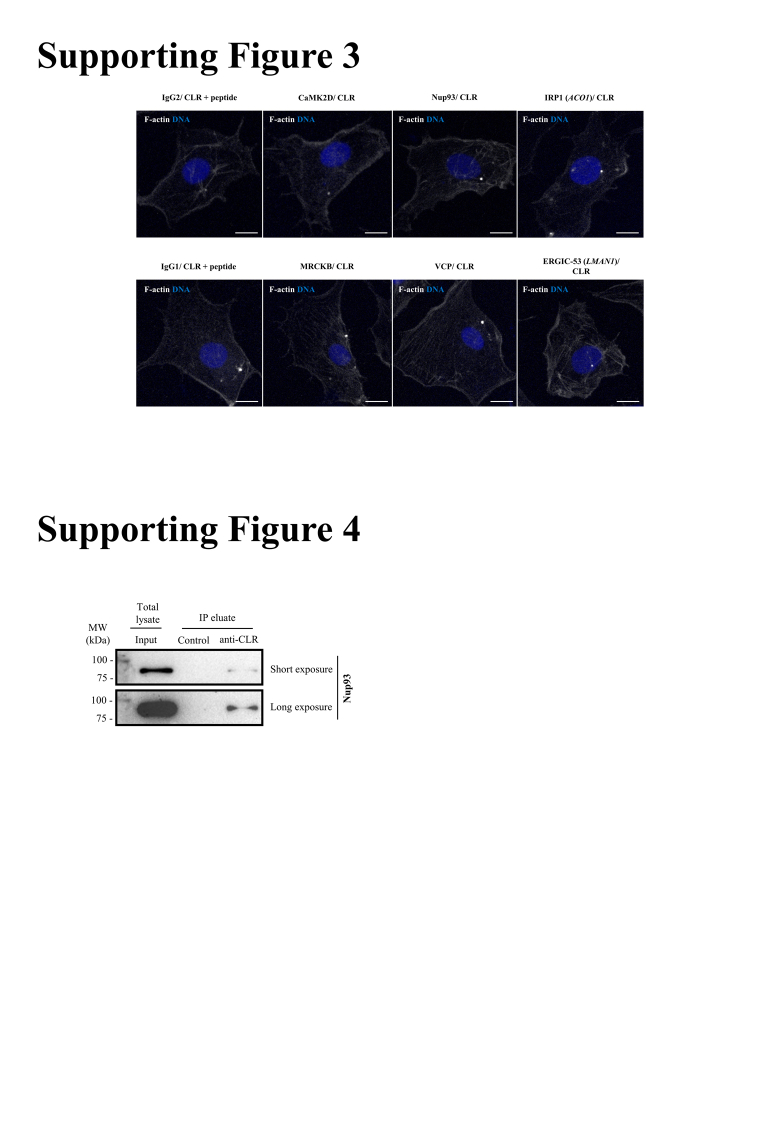# Quantitative proteomics reveals CLR interactome in primary human cells

**DOI:** 10.1016/j.jbc.2024.107399

**Published:** 2024-05-20

**Authors:** Dimitrios Manolis, Shirin Hasan, Anthony Maraveyas, Darragh P. O'Brien, Benedikt M. Kessler, Holger Kramer, Leonid L. Nikitenko

**Affiliations:** 1 Centre for Biomedicine, Hull York Medical School, University of Hull, Hull, UK; 2 Queens Centre for Oncology and Haematology, Castle Hill Hospital, Hull University Teaching Hospitals NHS Teaching Trust, Hull, UK; 3 Target Discovery Institute, Centre for Medicines Discovery, Nuffield Department of Medicine, University of Oxford, Oxford, UK; 4 Medical Research Council Laboratory of Molecular Biology, Cambridge, UK

**Keywords:** calcitonin receptor–like receptor, GPCR, endothelial cell, endogenous, interactome, proteome

## Abstract

The G protein–coupled receptor (GPCR) calcitonin receptor–like receptor (CLR) mediates essential functions in several cell types and is implicated in cardiovascular pathologies, skin diseases, migraine, and cancer. To date, the network of proteins interacting with CLR (“CLR interactome”) in primary cells, where this GPCR is expressed at endogenous (physiologically relevant) levels, remains unknown. To address this knowledge gap, we established a novel integrative methodological workflow/approach for conducting a comprehensive/proteome-wide analysis of *Homo sapiens* CLR interactome. We used primary human dermal lymphatic endothelial cells and combined immunoprecipitation utilizing anti-human CLR antibody with label-free quantitative nano LC-MS/MS and quantitative *in situ* proximity ligation assay. By using this workflow, we identified 37 proteins interacting with endogenously expressed CLR amongst 4902 detected members of the cellular proteome (by quantitative nano LC-MS/MS) and revealed direct interactions of two kinases and two transporters with this GPCR (by *in situ* proximity ligation assay). All identified interactors have not been previously reported as members of CLR interactome. Our approach and findings uncover the hitherto unrecognized compositional complexity of the interactome of endogenously expressed CLR and contribute to fundamental understanding of the biology of this GPCR. Collectively, our study provides a first-of-its-kind integrative methodological approach and datasets as valuable resources and robust platform/springboard for advancing the discovery and comprehensive characterization of physiologically relevant CLR interactome at a proteome-wide level in a range of cell types and diseases in future studies.

The G protein–coupled receptors (GPCRs) are the largest and most drug-targeted superfamily of membrane receptors in humans (1). GPCRs are activated by ligands to tightly regulate a wide range of physiological and pathological responses in a cell type– or tissue-specific manner (2, 3). A complex network of proteins interacting with GPCRs regulates ligand binding, localization to the plasma membrane, downstream signaling, and other properties and functions of these receptors (4, 5). These GPCR interactors belong to several protein classes including kinases, scaffold/adaptor proteins, transcription factors, and trafficking regulators, such as transporters and chaperones (6, 7).

The calcitonin receptor–like receptor (CL receptor or CLR) is a class B GPCR encoded by the *CALCRL* gene (8, 9). CLR is expressed and mediates essential functions in several cell types, including blood and lymphatic vessel endothelial cells (EC), vascular smooth muscle cells (VSMCs), cardiomyocytes, neurons, and cancer cells (10, 11, 12, 13, 14, 15). CLR and its three putative peptide agonists–adrenomedullin (AM), calcitonin gene-related peptide (CGRP), and AM2/intermedin are implicated in cardiovascular and skin diseases, migraine, and cancer (16, 17, 18, 19, 20, 21, 22, 23, 24, 25, 26). Targeting the CLR signaling axis for migraine prophylaxis has been associated with impaired wound healing, ischemic events, and side effects (27, 28, 29, 30, 31). Altogether, these findings warrant a detailed investigation of CLR properties in cells where this receptor is expressed and contributes to a range of physiological and pathological conditions.

Receptor overexpression studies using hemagglutinin (HA), Myc, or green fluorescent protein (GFP) tagging and transient or stable transfection methodological approaches in mammalian immortalized cell lines have provided some insights into the regulation of CLR activity by proteins interacting with this GPCR (32, 33, 34, 35, 36). For example, these reports demonstrated that glycosylation state, transportation from the endoplasmic reticulum (ER) to the plasma membrane, affinity for agonists; internalization and trafficking of CLR are determined by its interaction (heterodimerization) with receptor activity-modifying proteins (RAMP) 1, 2, and 3. Despite these findings and advances, the limitations of receptor overexpression approaches for studying CLR properties, which are significantly influenced by cell-specific factors, have been also widely acknowledged (37). In particular, there are some mismatches of results from studies investigating CLR pharmacology using receptor overexpression when compared to endogenous expression (nonoverexpressed or knocked out; and hence recapitulating best physiological relevance) in primary cells (37). Protein overexpression with or without tagging often leads to nonphysiological abundance when compared to endogenous levels and/or to interference with putative interactors (38). More specifically, it might alter the stoichiometry of protein–protein interactions, increasing their nonspecificity (false positive rate of identifications), affecting protein folding or modifications and, ultimately, cellular responses (39, 40).

The comprehensive characterization of the protein interaction network of CLR (herein termed “CLR interactome”) in primary cells, where this GPCR is expressed at endogenous levels, has the potential to unravel currently unknown mechanisms regulating the properties of this GPCR. To date, the knowledge about protein complexes associated with endogenously expressed CLR is limited to studies in *Cavia porcellus* (guinea pig) cerebellum tissue and *Mus musculus* NIH3T3 cells, where chaperone receptor component protein was coimmunoprecipitated with this receptor (41, 42). However, to our knowledge, there are no data regarding the physiologically relevant interactome of CLR expressed in primary cells from *Homo sapiens* or other species. This limits the progress of both fundamental and preclinical (translational) research in the field (43, 44, 45).

Three major challenges impede the purification of endogenously expressed GPCRs, including CLR, and the discovery of their physiologically relevant interactomes: (1) the low expression levels of these receptors (2), the lack of highly specific antibodies, and (3) the paucity in identification and quantification of GPCR interactions with other proteins in a “native” (without using chemical labeling/modification, which alters properties of the proteins and, subsequently, cell biology; *i.e.*, “label-free”) state (40, 46, 47, 48). To overcome these challenges, primary human dermal lymphatic endothelial cells (HDLECs) or other types of cells, in which CLR is endogenously expressed in abundance, can be used as a model for studying the properties, functions, and regulation of this GPCR (11, 13, 49). Furthermore, coimmunoprecipitation (co-IP) of GPCRs with mass spectrometry (MS)-based proteomics have been successfully utilized for the identification and characterization of novel GPCR-associated protein complexes, including for β2-adrenergic receptor (β2-AR) and others, but not CLR (50). Our anti-human CLR antibody has been extensively characterized (11), but not yet used in immunoprecipitation (IP) or co-IP studies, including the combination with quantitative MS-based (co-IP-MS) methodological approaches, for facilitating the discovery and quantitative analysis of CLR interactome in primary human cells and tissues (40). Moreover, while coupling of nano liquid chromatography with label-free quantitative tandem mass spectrometry (nano LC-MS/MS) provided sensitive detection, high coverage, and quantification of human EC proteome (51), this method is yet to be tested for the analysis of physiologically relevant CLR interactome. Finally, *in situ* proximity ligation assay (PLA) has emerged as a powerful tool for the detection and quantification of interactions of endogenously expressed GPCR in cells and tissues, including validation of co-IP findings (52).

In our first-of-its-kind study, we successfully developed and applied a novel (integrative and unique in its nature) methodological approach/workflow to comprehensively characterize the *H. sapiens* CLR interactome in primary human cells. We efficiently purified CLR endogenously expressed in HDLEC by IP using our anti-human CLR antibody and utilized a combination of state-of-the-art methods (label-free quantitative nano LC-MS/MS and quantitative *in situ* PLA) to discover the distinct network of 37 proteins interacting with this receptor and reveal direct interactions. Our study provides a conceptual advance over previous approaches for studying physiologically relevant CLR interactome and fundamental insights into the biology of this GPCR.

## Results

### Label-free quantitative nano LC-MS/MS reveals endogenously expressed CLR in the context of HDLEC cellular proteome

**Figure 1:**
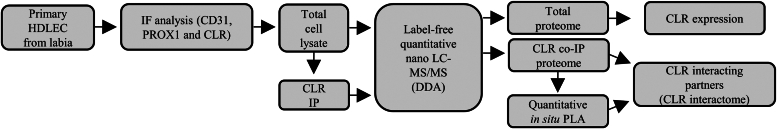
**Flow diagram of key components of the integrative approach/platform used to study physiologically relevant CLR interactome at a proteome-wide level in human primary cells.** Human dermal lymphatic endothelial cells (HDLEC) were characterized by analyzing the expression of pan-endothelial cell and lymphatic endothelial cells (LEC)-specific markers (cluster of differentiation 31 (CD31) and prospero homeobox protein 1 (PROX1), respectively) and calcitonin receptor–like receptor (CLR) by immunofluorescence (IF). Highly specific and well characterized by us previously anti-human CLR rabbit polyclonal antibody/immune serum LN-1436 (11, 17) was used for immunoprecipitation (IP). Preimmune serum obtained from the same rabbit (11, 17) was used for control IP. The analyses of the cellular and CLR co-IP proteomes were carried out by label-free quantitative nano liquid chromatography tandem mass spectrometry (nano LC-MS/MS) in data-dependent acquisition (DDA) mode. The validation of detected by nano LC-MS/MS interactions of CLR with other proteins was assessed by quantitative *in situ* proximity ligation assay (PLA).

**Figure 2:**
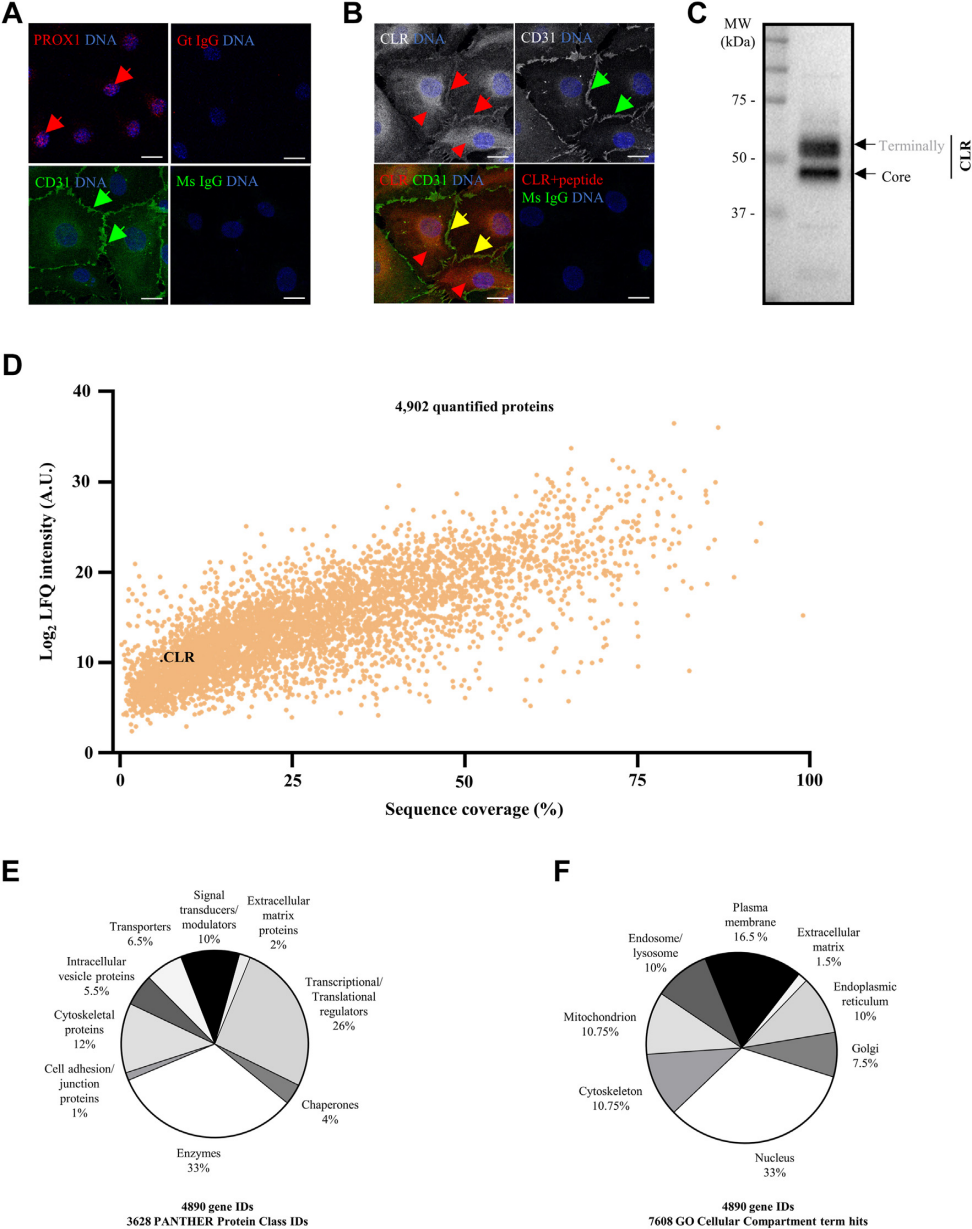
**Endogenous expression of CLR in the context of human dermal lymphatic endothelial cell proteome.***A* and *B*, immunofluorescence (IF) analysis of prospero homeobox protein 1, PROX1 (*red*), cluster of differentiation 31, CD31 (*green*), and calcitonin receptor–like receptor, CLR (*red*), expression in human dermal lymphatic endothelial cells (HDLECs) fixed using (*A*) paraformaldehyde or (*B*) acetone-methanol (2:3). IF for CLR was done using rabbit anti-human CLR antibody/immune serum LN-1436 (11), and preincubation with immunizing peptide (CLR + peptide) was used as a negative control for immunostaining (11, 17); Experimental procedures). Anti-mouse (Ms) or anti-goat (Gt) immunoglobulins (IgG) were used as isotype controls (*right panel* in *A* and *lower right side* image in *B*). *A*, PROX1 expression in the nucleus (*red arrows*) and CD31 expression at cell–cell contacts (*green arrows*). *B*, CLR (*red arrows*) and CD31 (*green arrows*) coexpression on cell membrane upon their colocalization (*yellow arrows*), and CLR expression intracellularly in the perinuclear space (*red arrowheads*). *A* and *B*, the scale bars represent 20 μm. *C*, immunoblotting (IB) analysis of core-glycosylated and terminally glycosylated forms of CLR endogenously expressed in HDLEC using anti-human CLR antibody/immune serum LN-1436 (11). *D*–*F*, label-free quantitative nano liquid chromatography-tandem mass spectrometry (nano LC-MS/MS) analysis of HDLEC lysates. The summary results of four independent experiments are presented. The raw MS files and search/identification files obtained with MaxQuant have been deposited to the ProteomeXchange Consortium *via* the PRIDE partner repository (102) with the dataset identifier PXD032156. *D*, *scatter plot* of HDLEC proteome, in which the percentage of sequence coverage is plotted against the log_2_ label-free quantitation (LFQ) intensity for each quantified protein. Each dot (*orange color*) in the *scatterplot* represents a quantified protein and CLR is *highlighted* (*black dot*). 56,930 peptides and 5102 proteins groups were identified and 4902 protein groups were quantified. Full list is presented in Table S1. *E* and *F*, *pie charts* reflecting the results of Protein ANalysis THrough Evolutionary Relationships (PANTHER) Protein Class and Gene Ontology (GO) cellular compartment (CC) analyses of quantified HDLEC proteome. GO terms were mapped using the PANTHER classification system (113, 114). Percentage of (*E*) different protein classes (mapping 3628 PANTHER protein class IDs from 4890 gene identifiers) and (*F*) subcellular localization (mapping of 7608 hits of GO CC terms from 4890 genes identifiers) to which the members of HDLEC proteome belong. MS, mass spectrometry.

To investigate CLR interactome in primary human cells at proteome-wide level, we established a novel methodological approach/workflow by using HDLEC as a cell model, IP with well-characterized anti-human CLR antibody/immune serum LN-1436 (11) and a combination of quantitative MS and non-MS–based approaches (Fig. 1). Immunofluorescence (IF) and immunoblotting analyses demonstrated that a pure population of HDLEC expressed core-glycosylated and terminally glycosylated CLR forms, with receptor localization in perinuclear space and at the cell surface (Fig. 2, *A*–*C*). Next, a comprehensive label-free quantitative LC-MS/MS analysis of total cell lysates generated a large-scale proteome profile of primary HDLEC consisting of 4902 proteins (cellular proteome), including CLR (Fig. 2*D* and Table S1). According to Gene Ontology (GO) analysis using Protein ANalysis THrough Evolutionary Relationships (PANTHER), the HDLEC proteome includes enzymes (33%), transcriptional/translational regulators (26%), signal transducers/modulators (10%), and molecules from other protein classes (Fig. 2*E*), which are associated with various cellular compartments (Fig. 2*F*).

Next, CLR abundance was quantified in the context of HDLEC total cell lysates/cellular proteome based on obtained data about two identified peptides covering 6.7% of its sequence (Tables 1 and S1). The proteomic ruler analysis (53) revealed the relative abundance (based on protein copy numbers and abundance in molecules and mass) of endothelial markers together with transmembrane receptors in primary HDLEC (Tables 1 and 2). This analysis revealed that CLR abundance was the highest when compared to selected lymphatic-specific and pan-endothelial markers and receptors, such as prospero homeobox protein 1, cluster of differentiation 31, vascular endothelial growth factor receptor 3, and others (Table 1). Furthermore, proteome profiling followed by quantitative analysis of MS-based data identified 34 transmembrane receptors, including 11 GPCRs and nine receptor tyrosine kinases (Table 2).

**Table 1:** Summary of expression levels of selected pan-endothelial and lymphatic-specific endothelial cell markers in human dermal lymphatic endothelial cells in the context of total proteome

Gene	Protein (UniProtKB-Swiss-Prot ID)	UniProtKB-Swiss-Prot ID	Mean copy number	Mean abundance (molecules/total molecules) [∗10^∧^-6]	Mean abundance (mass/total mass) [∗10^∧^-6]	Peptide to spectra matches (N)	Mean log2 LFQ intensity (A.U.)	Peptides (N)	Sequence coverage [%]
*CALCRL*	Calcitonin gene-related peptide type 1 receptor or calcitonin receptor–like receptor	Q16602	7.8E+06	285.5	398.6	24	25.2	2	6.7
*LYVE1*	Lymphatic vessel endothelial hyaluronic acid receptor 1	Q9Y5Y7	6.0E+06	217.1	201.7	21	24.9	2	5.6
*CDH5*	Cadherin-5	P33151	3.9E+06	142.6	329.2	202	30.2	18	31.9
*CD31/PECAM1*	Cluster of differentiation 31/platelet endothelial cell adhesion molecule 1	P16284	2.2E+06	80.5	175.3	446	32.0	34	48.1
*PROX1*	Prospero homeobox protein 1	Q92786	2.0E+06	73.5	161.4	19	24.7	6	10.3
*TIE1*	Tyrosine-protein kinase receptor Tie-1	P35590	1.7E+06	61.8	204.0	51	27.3	11	10
*FLT4*	Vascular endothelial growth factor receptor 3	P35916	1.4E+06	49.5	191.1	45	26.9	14	15.3
*KDR*	Vascular endothelial growth factor receptor 2	P35968	1.2E+06	43.0	171.9	21	25.1	8	7.9
*VWF*	von Willebrand factor	P04275	7.5E+05	27.5	224.7	722	32.4	60	24.9

The relative abundance in protein copy numbers, molecules per total molecules, mass per total mass together with peptide to spectra matches, mean log_2_ label-free quantitation (LFQ) intensity, number of identified peptides, and percentage of sequence coverage for pan-endothelial or lymphatic endothelial cell-specific markers in human dermal lymphatic endothelial cells (HDLECs) lysates are listed. The summary results of four independent experiments are presented. Protein copy numbers and abundance in molecules and mass were estimated using the proteomic ruler tool (53). Proteins are listed in order of the highest to lowest copy number. Calcitonin receptor-like receptor (CLR; *CALCRL*) is highlighted in gray.

**Table 2:** Quantitative analysis of expression levels of transmembrane receptors in human dermal lymphatic endothelial cells in the context of cellular proteome

Receptor family	Gene	Protein	UniProtKB-Swiss-Prot ID	Mean copy number	Mean abundance (molecules/total molecules) [∗10^∧^−6]	Mean abundance (mass/total mass) [∗10^∧^−6]	Peptide to spectra matches (N)	Mean log_2_ LFQ intensity (A.U.)	Peptides (N)	Sequence coverage [%]
G protein–coupled receptor	*SSR3*	Somatostatin receptor type 3	Q9UNL2	1.81E+07	660.1	367.1	24	29.2	2	11.9
	*SSR4*	Somatostatin receptor type 4	P51571	1.44E+07	526.5	263.9	82	30.2	5	36.4
	*SSR1*	Somatostatin receptor type 1	P43307	8.66E+06	316.0	270.5	80	29.3	6	28.1
	*CALCRL*	Calcitonin gene-related peptide type 1 receptor or calcitonin receptor-like receptor	Q16602	7.83E+06	285.5	398.6	24	25.2	2	6.7
	*ADGRG1*	Adhesion G-protein coupled receptor G1	Q9Y653	6.92E+06	252.6	140.0	12	22.3	1	5.3
	*GPRC5B*	G-protein coupled receptor family C group 5-member B	Q9NZH0	6.27E+06	226.4	267.6	9	24.0	2	6
	*F2R*	Proteinase-activated receptor 1	P25116	5.48E+06	199.8	250.0	14	22.9	1	4.2
	*S1PR1*	Sphingosine 1-phosphate receptor 1	P21453	4.66E+06	170.1	192.1	10	24.0	3	12.6
	*ADGRL4*	Adhesion G protein–coupled receptor L4	Q9HBW9	3.26E+06	119.1	127.5	51	27.4	6	20.5
	*ADGRE5*	Adhesion G protein–coupled receptor E5	P48960	2.38E+06	86.7	210.0	16	23.0	3	6.3
	*ADGRF5*	Adhesion G protein–coupled receptor F5	Q8IZF2	1.40E+06	50.9	200.8	48	26.1	6	6.4
Receptor protein tyrosine kinases	*AXL*	Tyrosine-protein kinase receptor UFO	P30530	1.86E+06	68.9	178.8	13	25.6	6	8.6
	*EPHA2*	Ephrin type-A receptor 2	P29317	1.85E+06	67.5	192.7	141	28.0	17	22
	*TEK*	Angiopoietin-1 receptor	Q02763	1.72E+06	62.9	208.6	82	27.2	14	16.9
	*TIE1*	Tyrosine-protein kinase receptor Tie-1	P35590	1.69E+06	61.8	204.0	51	27.3	11	10
	*EPHB4*	Ephrin type-B receptor 4	P54760	1.65E+06	60.3	172.2	144	28.2	18	23.7
	*EPHB2*	Ephrin type-B receptor 2	P29323	1.61E+06	58.7	181.8	31	25.4	7	9
	*FLT4*	Vascular endothelial growth factor receptor 3	P35916	1.36E+06	49.5	191.1	45	26.9	14	15.3
	*MET*	Hepatocyte growth factor receptor	P08581	1.21E+06	44.2	181.3	69	26.2	11	9.5
	*KDR*	Vascular endothelial growth factor receptor 2	P35968	1.18E+06	43.0	171.9	21	25.1	8	7.9
Cytokine receptor	*IL6ST*	Interleukin-6 receptor subunit beta	P40189	2.13E+06	77.8	212.4	153	28.8	19	28.6
Tumor Necrosis Factor receptor	*TNFRSF10C*	Tumor necrosis factor receptor superfamily member 10C	O14798	8.74E+06	315.4	227.9	15	22.3	1	5.8
	*TNFRSF10B*	Tumor necrosis factor receptor superfamily member 10B	O14763	4.47E+06	163.0	205.9	34	24.5	3	9.5
	*TNFRSF10A*	Tumor necrosis factor receptor superfamily member 10A	O00220	3.75E+06	135.4	121.1	3	24.7	2	7.4
	*FAS*	Tumor necrosis factor receptor superfamily member 6	P25445	3.74E+06	136.4	135.8	17	24.1	2	8.4
Other transmembrane receptor	*SIGMAR1*	Sigma non-opioid intracellular receptor 1	Q99720	1.58E+07	577.7	382.9	13	25.5	5	35.4
	*CD44*	CD44 antigen	P16070	7.49E+06	273.2	163.5	109	29.0	8	43.2
	*LYVE1*	Lymphatic vessel endothelial hyaluronic acid receptor 1	Q9Y5Y7	5.95E+06	217.1	201.7	21	24.9	2	5.6
	*ENG*	Endoglin	P17813	4.94E+06	180.3	335.7	148	30.3	12	29.5
	*GRID2*	Glutamate receptor ionotropic, delta-2	O43424	2.03E+06	73.9	203.1	6	28.4	1	1.2
	*NID1*	Nidogen-1	P14543	1.70E+06	61.9	222.5	117	29.0	21	21.6
	*PLXND1*	Plexin-D1	Q9Y4D7	1.10E+06	40.0	223.7	370	29.7	39	22.6
	*PLXNA2*	Plexin-A2	O75051	9.13E+05	33.3	185.5	153	27.4	21	13.4
	*PLXNB2*	Plexin-B2	O15031	8.89E+05	32.4	175.6	30	25.8	13	9.7

The relative abundance of identified in our study transmembrane receptors in human dermal lymphatic endothelial cell (HDLEC) lysates was assessed and listed by mean protein copy numbers, molecules per total molecules, mass per total mass together with peptide to spectra matches, mean log_2_ label-free quantitation (LFQ) intensity, number of identified peptides, and percentage of sequence coverage. The summary results of four independent experiments are presented. Protein copy numbers and abundance in molecules and mass were estimated using the proteomic ruler tool (53). Proteins are listed in order of the highest to lowest copy number. All listed G protein–coupled receptors, except calcitonin receptor–like receptor (CLR; *CALCRL*) have not been previously reported in *in vitro* cultured HDLEC (n = 10).

### Core-glycosylated and terminally glycosylated forms of endogenously expressed CLR can be efficiently immunoprecipitated from primary human cells

**Figure 3:**
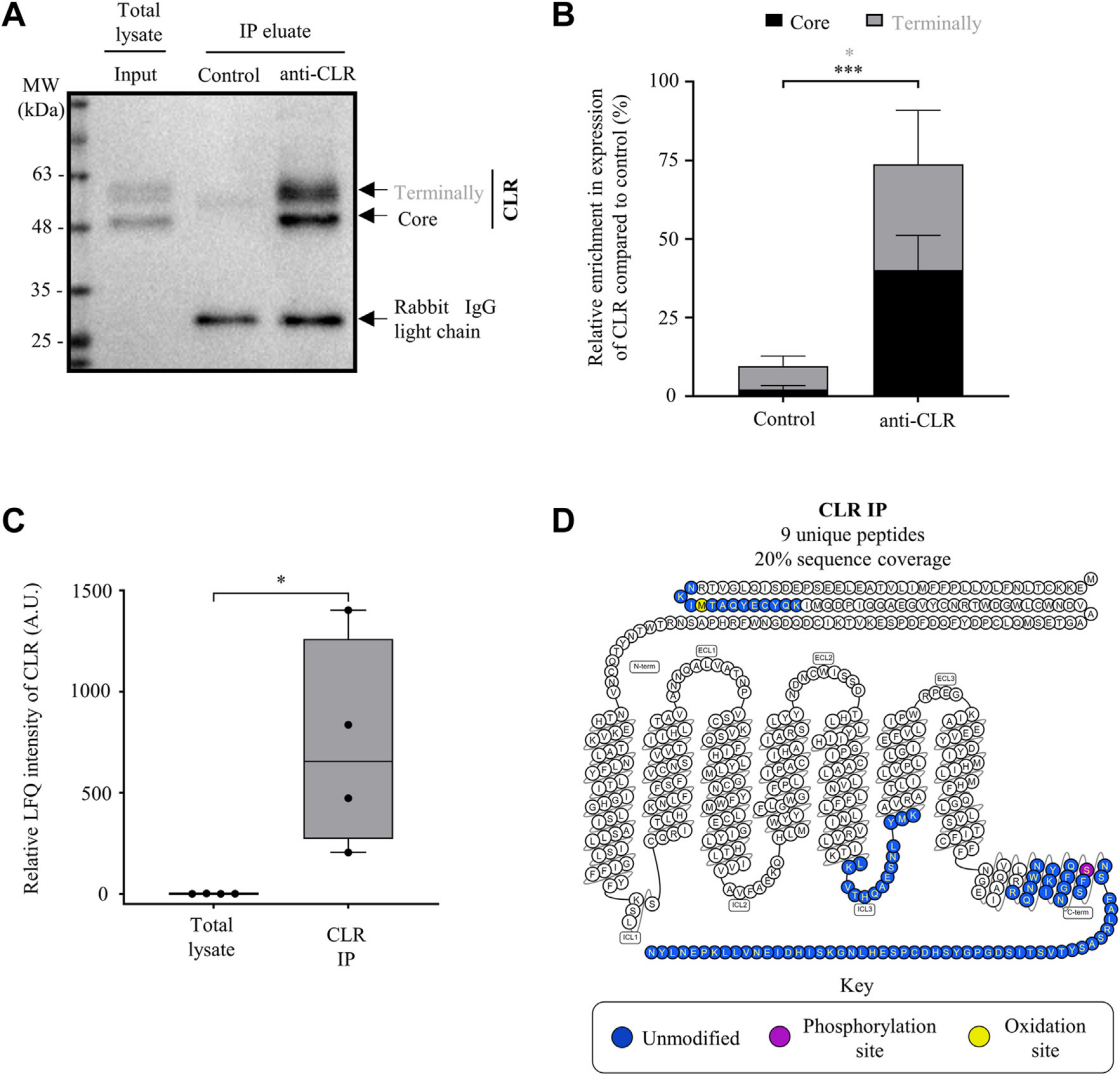
**Immunoprecipitation of CLR endogenously expressed in human dermal lymphatic endothelial cells.***A* and *B*, immunoprecipitation (IP) of CLR from total cell lysates/cellular proteome (input) of *in vitro* cultured human dermal lymphatic endothelial cells (HDLEC) and immunoblotting were performed using anti-human calcitonin receptor–like receptor (CLR) antibody/immune serum LN-1436 (11). Preimmune serum served as a negative control and detection was done using anti-rabbit IgG light chain antibody. Detection (*A*) and quantification (*B*) of CLR expression in IP samples. *B*, the data represents the mean ± SD for expression of core-glycosylated (40.2% ± 9.45) and terminally glycosylated (33.5% ± 14.87) forms of CLR upon IP, compared to expression of core-glycosylated form in control group (four independent experiments; D'Agostino-Pearson (*p* > 0.05); unpaired Student’s *t* test; ∗*p* < 0.05, ∗∗∗*p* < 0.001). *C*, relative abundance of CLR in IP samples compared to total cell lysates/cellular proteome (n = 4 independent biological replicates; *box and whiskers plot* represents median values). The box contains the 25th and 75th percentiles and whiskers are the minimum and maximum LFQ values of each dataset. The statistical analysis was performed using Shapiro–Wilk (*p* < 0.05), followed by Mann–Whitney test; ∗*p* < 0.05). The calculations are based on the log_2_ label-free quantitation (LFQ) intensity values acquired upon normalization of raw label-free quantitative nano LC-MS/MS data (peptide intensities) of all identified proteins between the two datasets (Experimental procedures; Fig. S1), followed up by normalization of CLR IP against the total lysate. *D*, *snake plot* generated by using GPCRdb (115) representing sequence coverage (20%) of CLR based on the peptides identified (key; *colored amino acids*) in our study. C term, C terminus; ECL, extracellular loop; ICL, intracellular loop; N term, N terminus.

Next, we immunoprecipitated CLR from HDLEC total cell lysates/cellular proteome and analyzed IP samples by immunoblotting and label-free quantitative nano LC-MS/MS (Fig. 3). Immunoblotting analysis demonstrated that both forms (core-glycosylated and terminally glycosylated) of this GPCR were enriched with high efficiency upon IP (Fig. 3, *A* and *B*). To the best of our knowledge, this is the first report of successful IP of endogenously expressed human CLR.

**Figure S1:**
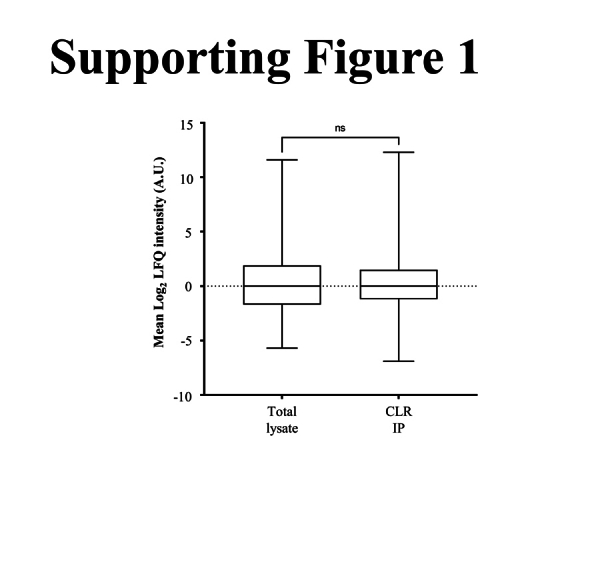


Next, we conducted label-free quantitative nano LC-MS/MS of IP samples (Fig. 1), normalized raw LC-MS/MS data (peptide intensities) between CLR IP and total cell lysate samples to obtain label-free quantitation (LFQ) intensity values for all identified proteins in both sets (Fig. S1) and then analyzed CLR abundance (Fig. 3*C*). Following IP-assisted enrichment, CLR abundance was increased by 534-fold on average (between 200- and 1400-fold change) in IP when compared to total cell lysates/cellular proteome samples (Fig. 3*C*).

Furthermore, nine CLR peptides were identified in HDLEC upon IP, seven more than in total cell lysates/cellular proteome detected in our study, and five more compared to a study in which EC isolated from human skin tissues and data-independent acquisition were used (54) (Table 3). These nine peptides are located in the receptor’s N terminus, third intracellular loop, and C terminus, covering 20% of the CLR sequence (Fig. 3*D*). To our knowledge, this is the highest sequence coverage for CLR (in *H. sapiens* or other species) reported in a single MS-based study to date. Therefore, our results indicate that efficient IP of both core-glycosylated and terminally glycosylated forms of endogenously expressed CLR was achieved, generating a solid foundation for defining the physiologically relevant interactome of this GPCR.

**Table 3:** Abundance of CLR peptides in cultured human dermal lymphatic endothelial cells compared to endothelial cells isolated from human skin

Domain	Peptide sequence	Modification	Peptide to spectra matches per million spectra		
			Current study (identifier PXD032156)		Dyring-Andersen *et al.*, 2020 (identifier PXD019909)
			IP	Total lysate	Total lysate
N terminus	NKIMTAQYECYQK	Unmodified	190	0	0
		Oxidation (M)	81	0	0
	IMTAQYECYQK	Unmodified	0	0	3
		Oxidation (M)	27	0	9
	ICDQDGNWFR	Unmodified	0	0	6
ICL3	LKVTHQAESNLYMK	Unmodified	108	0	0
	VTHQAESNLYMK	Unmodified	108	0	0
C terminus	IQFGNSFSNSEALR	Unmodified	217	22	9
		Phosphorylation (S)	54	0	0
	NWNQYKIQFGNSFSNSEALR	Unmodified	108	0	0
	RNWNQYKIQFGNSFSNSEALR	Unmodified	108	0	0
	SASYTVSTISDGPGYSHDCPSEHLNGK	Unmodified	81	0	13
	SIHDIENVLLKPENLYN	Unmodified	190	14	6
	SIHDIENVLLK	Unmodified	0	0	6

List of calcitonin receptor–like receptor (CLR) peptides identified by label-free quantitative nano liquid chromatography-tandem mass spectrometry. Domain, amino acid sequences, modifications, and peptide-spectra matches (per million spectra) of each peptide are also shown. Peptide to spectra matches acquired using immunoprecipitation samples or total cell lysates of *in vitro* cultured human dermal lymphatic endothelial cells (HDLECs) in the current study, compared to endothelial cells isolated from human skin tissue in a separate study (54). Respective identifiers of deposited datasets (PXD032156; PXD019909) at the ProteomeXchange Consortium *via* the PRIDE partner repository (102) are indicated.

### Label-free quantitative nano LC-MS/MS reveals the first comprehensive interactome of *H. sapiens* CLR

**Figure 4:**
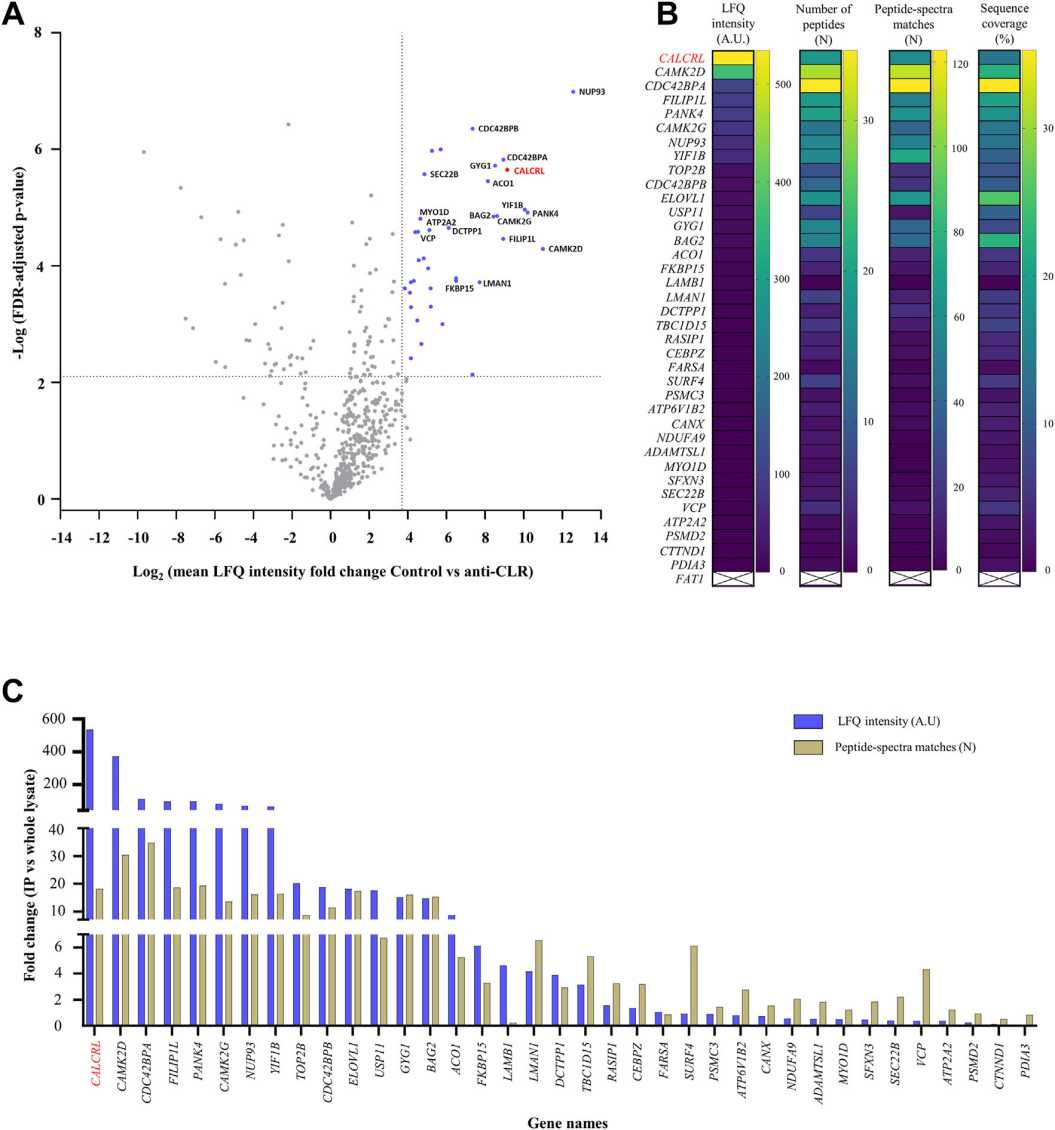
**Identification of proteins interacting with CLR endogenously expressed in human dermal lymphatic endothelial cells.***A*–*C*, identification and quantification of proteins coimmunoprecipitated with calcitonin receptor–like receptor (CLR) expressed in human dermal lymphatic endothelial cell (HDLEC) lysates by using label-free quantitative nano liquid chromatography-tandem mass spectrometry (nano-LC-MS/MS). *A*, *volcano plot* of nano-LC-MS/MS data showing the magnitude of difference in abundance (represented by log_2_ label-free quantitation (LFQ) intensity), plotted against the significance (showed by -log_10_ false discovery rate (FDR)-adjusted *p* values, derived by using *t* test), for each protein for four independent experiments comparing CLR IP and/or control IP samples. The log_2_ LFQ intensity values were acquired upon normalization of raw label-free quantitative nano LC-MS/MS data (peptide intensities) of all identified proteins between CLR IP and control IP samples (Experimental procedures). *Vertical dotted lines* denote absolute fold change in LFQ intensity ≥3.5 (mean + 2 SD) and horizontal ones denote FDR-adjusted *p* value < 0.0075 (two-tailed unpaired Student’s *t* tests; FDR = 0.01; S0 = 0; -log_10_ FDR-adjusted *p* value = 2.13). CLR (encoded by *CALCRL* gene; *red dot* and *red color*) and its interacting partners with the highest significance are labeled (37 proteins; *blue dots*; gene names are indicated for top 20 enriched proteins; Table S3). Note that both anti-human CLR/immune serum LN-1436 and preimmune serum (control) were obtained from the same rabbit (11). Also, prior to statistical analysis of IP data, potential bead cytoplasmic and nuclear contaminants (as previously described (108)) were identified in both CLR and control IP samples (Table S2) and removed. *B*, heatmaps showing the relative (to HDLEC cellular proteome) fold-change in LFQ intensity, number of identified peptides, peptide-spectra matches, and percentage of sequence coverage of CLR (*red color*) and its 37 interacting partners identified upon co-IP. Prior to this analysis, raw LC-MS/MS data (peptide intensities) between CLR IP and total cell lysate samples to obtain LFQ intensity values for all identified proteins in both sets (see Fig. S1). Protocadherin Fat 1 (*FAT1*), elongation of very long-chain fatty acids protein 1 (*ELOVL1*), and ADAMTS like 2 (*ADAMTSL2*) indicated at the *bottom* (*uncolored*) were coimmunoprecipitated with CLR even though they were not identified in HDLEC proteome. Protocadherin FAT1 was only identified in IP samples and not in HDLEC total cell lysates. *C, bar chart* showing relative (to HDLEC cellular proteome) fold change in the number of identified peptides and LFQ intensity of CLR (*red color*) and its interacting partners upon co-IP. Co-IP, coimmunoprecipitation.

Next, we performed label-free quantitative nano LC-MS/MS analysis of CLR IP samples to identify proteins which interact with endogenously expressed CLR and therefore constitute the first comprehensive physiologically relevant interactome of this GPCR (Fig. 4*A* and Table S3). To achieve this, we evaluated the enrichment of these proteins using two levels of control/comparative analyses between CLR IP and control IP samples and between CLR IP and HDLEC total cell lysates/cellular proteome.

Firstly, we normalized raw LC-MS/MS data (peptide intensities) between CLR IP and control IP samples to obtain LFQ intensity values for all identified proteins and then analyzed statistically significant differences in abundance of these proteins between the two sets (CLR IP and control IP; Fig. 4*A* and Table S3). This analysis showed that CLR was detected only in CLR IP and not in control IP (Table S4) and coimmunoprecipitated with 37 proteins which were highly enriched in CLR IP *versus* control IP (“high confidence” interactors) in all four independent IP experiments (false discovery rate [FDR]-adjusted *p* value = 2.24 × 10^−6^; Fig. 4*A* and Table S3). All identified CLR interactors have not been previously reported.

Secondly, we compared the abundance (represented by LFQ intensity values) of CLR and its interactors in CLR IP samples to HDLEC total cell lysates/cellular proteome (after relevant normalization of label-free quantitative nano LC-MS/MS data; Fig. S1). The relative enrichment (compared to cellular proteome) of 37 novel members belonging to CLR interactome was examined by the fold change in LFQ intensity, number of identified peptides, number of peptide-spectrum matches, and percentage of sequence coverage (Fig. 4, *B* and *C*). CLR was identified as the protein with the highest (534-fold on average; between 200- and 1400-fold) increase in abundance, when compared to 37 “high-confidence” interactors (Fig. 4*C*). Among these proteins, calcium/calmodulin-dependent protein kinase 2 delta (CaMK2D and calcium/calmodulin-dependent protein kinase 2 gamma; encoded by *CAMK2D* and calcium/calmodulin-dependent protein kinase 2 gamma genes), cell division control protein 42 (CDC42) binding protein kinase alpha and beta (or myotonic dystrophy kinase-related CDC42-binding kinase alpha; MRCKA and MRCKB; *CDC42BPA* (cell division control protein 42 binding protein kinase alpha) and *CDC42BPB* (*cell division control protein 42 binding protein kinase beta*)), filamin A–interacting protein 1 like (FILIP1L; *FILIP1L*), pantothenate kinase 4 (PanK4; *PANK4*), nucleoporin 93 (Nup93; *NUP93*), and BAG cochaperone 2 (Bag2; *BAG2*) were enriched in CLR IP samples for all four analyzed parameters (Fig. 4*B*).

### *In situ* PLA reveals direct interactions of CLR with kinases and regulators of protein quality control and trafficking

**Figure 5:**
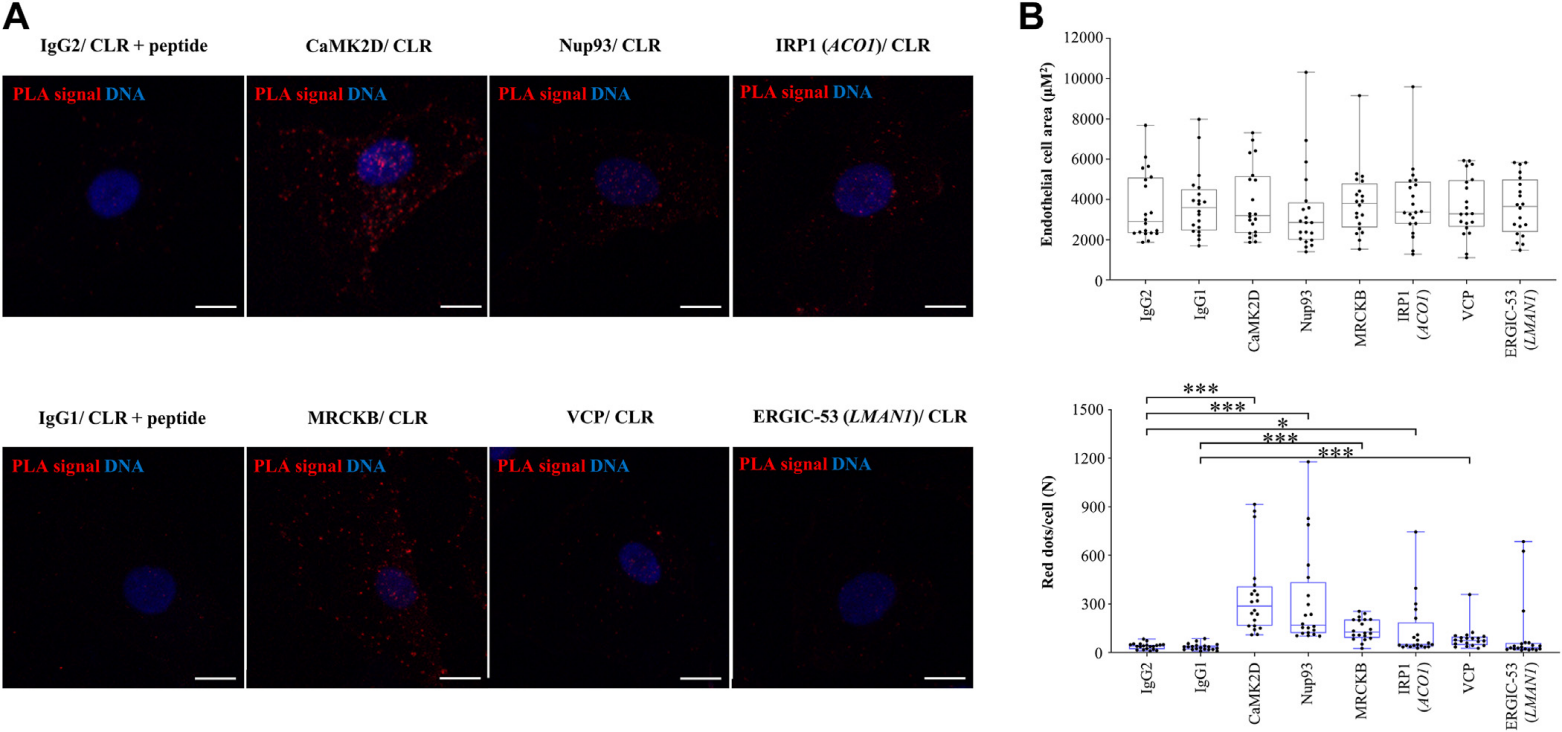
**Quantification of direct interactions of CLR endogenously expressed in human dermal lymphatic endothelial cells.***A* and *B*, *in situ* proximity ligation assay (PLA) was performed on paraformaldehyde-fixed human dermal lymphatic endothelial cells (HDLEC) to detect and quantify interactions of calcitonin receptor–like receptor (CLR) with highly enriched members of CLR interactome, as revealed by label-free quantitative nano liquid chromatography-tandem mass spectrometry (Fig. 4). Primary mouse mAbs were tested alongside relevant controls (Fig. S2) before being used in combination with anti-human CLR antibody/immune serum LN-1436 (11) for *in situ* PLA. *A*, representative images of PLA signal (*red dots*) for six proteins coimmunoprecipitated with CLR in HDLEC (see Fig. 4). See also Alexa Fluor 635 phalloidin (*white*; detecting F-actin) images of the same cells in Fig. S3. Nuclei were counterstained with DAPI (*blue*). The scale bars represent 10 μm. *B*, *box and whiskers plots* overlaid with *dot plots* represent the results (median) of quantification analysis of endothelial cell area in μm^2^ (*top*; see Fig. S3) and PLA signals (*red dots*) per cell (*bottom*) for six analyzed proteins (n = 20 cells per group). For proteins IRP1 and ERGIC-53, gene names are also indicated in *parentheses*. The box contains the 25th and 75th percentiles and whiskers are the minimum and maximum values for endothelial cell area (*B*-*top*; Shapiro–Wilk [*p* < 0.05]), followed by Kruskal–Wallis, or Shapiro–Wilk (*p* > 0.05), followed by unpaired Student’s *t* test; (data not significant) and PLA signal (*B*-*bottom*; Shapiro–Wilk (*p* < 0.05), followed by Mann–Whitney test, or Shapiro–Wilk (*p* > 0.05), followed by unpaired Student’s *t* test; ∗*p* < 0.05; ∗∗∗*p* < 0.001. DAPI, 4′,6-diamidino-2-phenylindole; ERGIC, ER-Golgi intermediate compartment; IRP1, iron regulatory protein 1.

**Figure S2:**
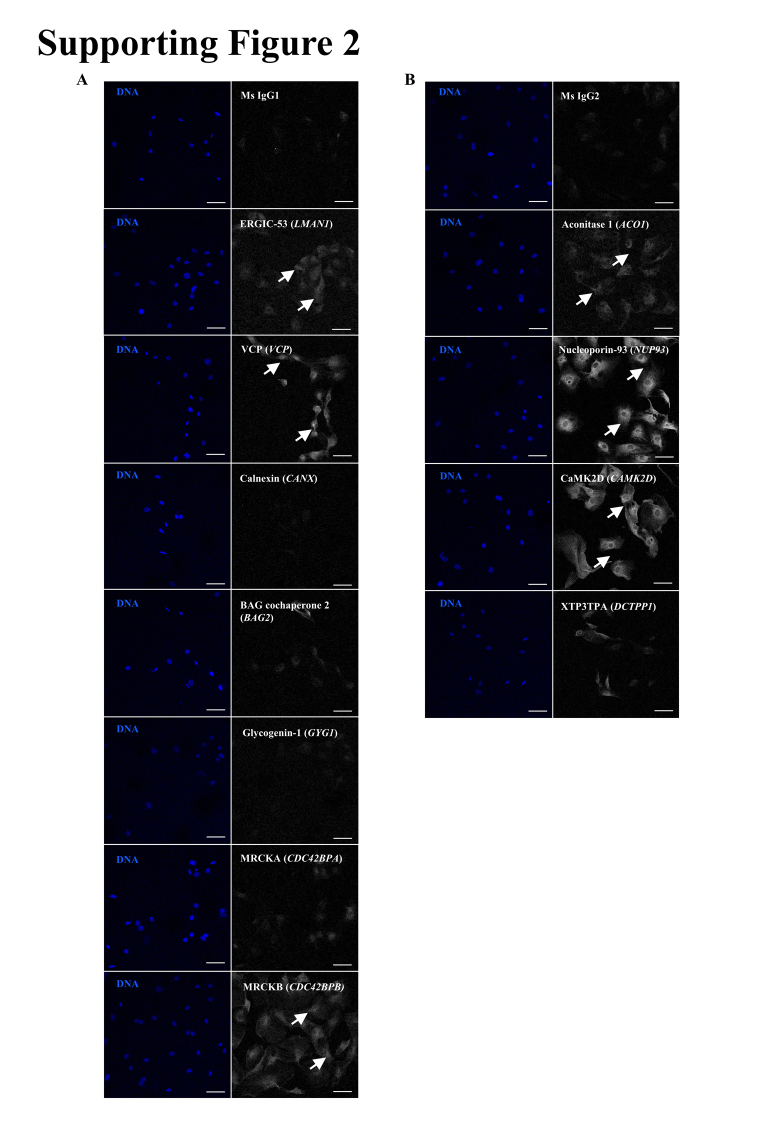


Next, 11 out of 37 proteins identified to interact with endogenously expressed CLR were selected for the validation of label-free quantitative nano LC-MS/MS findings by quantitative *in situ* PLA (Fig. S2). The detectable expression of CaMK2D, Nup93, MRCKB, iron regulatory protein 1 (IRP1; *ACO1*), valosin-containing protein (VCP or p97; *VCP*) and ER-Golgi intermediate compartment (ERGIC) marker 53 (ERGIC-53; *LMAN1*) in HDLEC allowed the identification and quantification of their direct interactions with endogenously expressed CLR by *in situ* PLA (Figs. 5, *A* and *B*; S3). Importantly, quantified *in situ* PLA signals for these members of CLR interactome agreed with findings from label-free quantitative LC-MS/MS analysis (compare Figs. 4*C* and 5*B*). For a second line of validation of LC-MS/MS findings, we focused on Nup93, as the most enriched protein in all four independent CLR IP when compared to control IP samples (∼12.6-fold difference; FDR-adjusted *p* value = 1.03 × 10^−7^; Table S3). Immunoblotting analysis detected Nup93 in CLR IP and not in control IP (Fig. S4).

### CLR interactome is a distinct network of proteins associated with GPCR function and regulation

**Figure 6:**
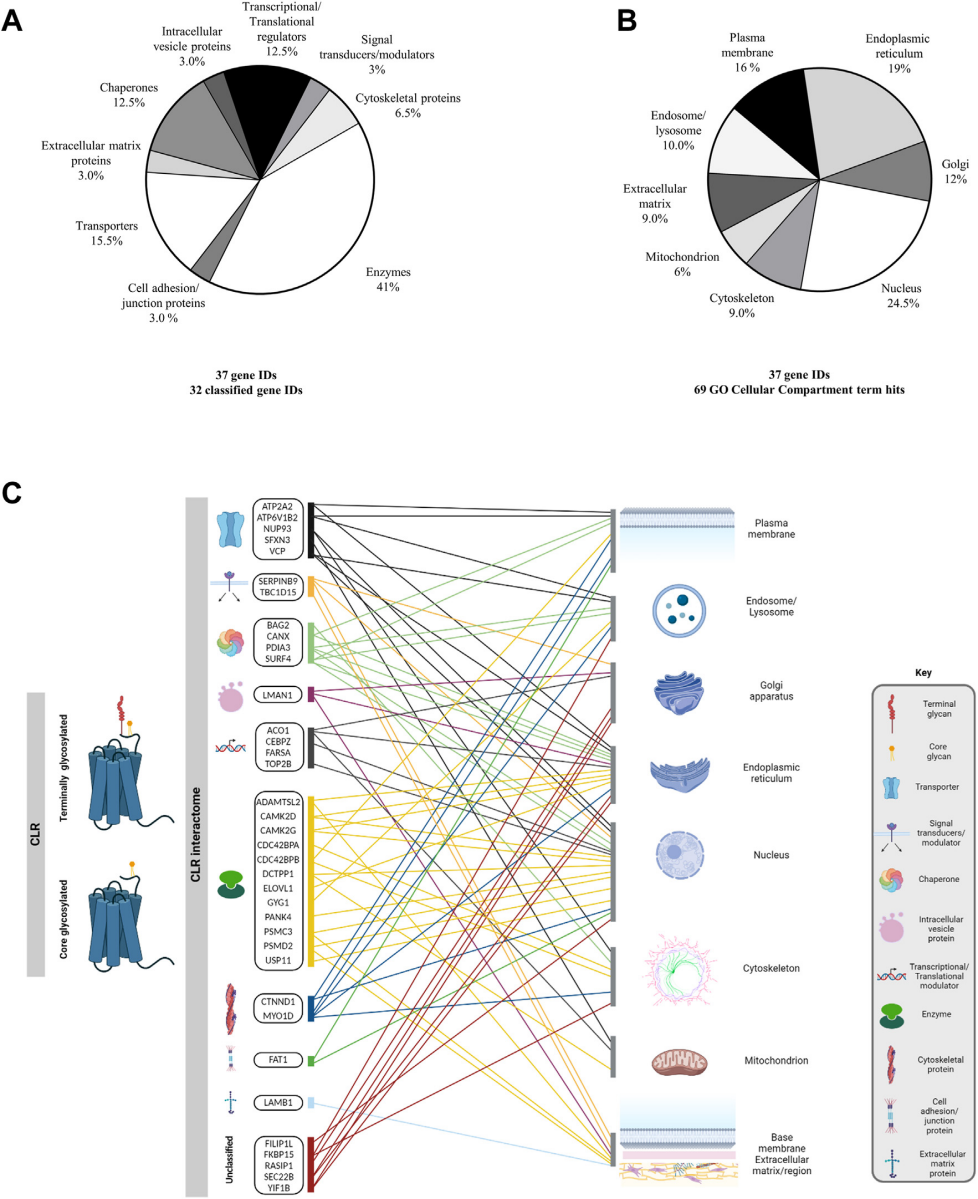
**Classification and sub-cellular localization of proteins belonging to physiologically relevant *Homo sapiens* CLR interactome.***A* and *B*, Protein ANalysis THrough Evolutionary Relationships (PANTHER) protein class and Gene Ontology (GO) cellular compartment (CC) analyses of 37 identified in this study members of calcitonin receptor–like receptor (CLR) interactome in human dermal lymphatic endothelial cells (HDLECs). GO terms were mapped using the PANTHER classification system (113, 114). Percentage of (*A*) different protein classes (mapping of 39 PANTHER protein class identifiers from 37 gene identifiers) and (*B*) subcellular localization (mapping of 89 GO CC term hits from 37 gene identifiers; Table S5). *C*, scheme representing PROTEIN CLASSes and predicted subcellular localization of 37 newly identified in our study members of CLR interactome (Fig. 4, *A* and *B*; Table S5). The protein classes are represented by specific icons (Key). Each protein (genes are indicated) is connected (*lines*) with its relevant cellular component (defined by GO classification), reflecting its predicted subcellular localization. The scheme created with BioRender.com.

Following confirmation by *in situ* PLA, comprehensive mapping of protein classes and subcellular localization of identified CLR interactome was conducted using PANTHER protein class and GO cellular compartment analyses (Fig. 6, *A*–*C* and Table S5). The proteins interacting with endogenously expressed CLR are associated with ER, intracellular vesicles, nucleus, and plasma membrane (Fig. 6*A* and Table S5). Importantly, our data showed that immunoprecipitated core-glycosylated and terminally glycosylated forms of CLR endogenously expressed in primary cells (Fig. 2*C*) interact with a larger network of proteins than it was previously described in studies using receptor overexpression models. This network includes a distinct subset of molecules that play roles in GPCR biology, such as specific guanine nucleotide exchange factors (55), chaperones (56), ubiquitin-specific proteases (57), nucleoporins (58), ER calcium ATPases (59), clathrin (60) and calcium/calmodulin-dependent protein kinases (61) (Fig. 6, *B* and *C*; Table S5).

## Discussion

The need to elucidate the interaction network of GPCRs, including CLR, in human cells and tissues has increased recently (Kotliar *et al.*, 2023). Although CLR and its agonists are involved in a wide range of disease states, the knowledge about the physiologically relevant interactome of this GPCR is limited. In our first-of-its-kind study, we applied a combination of co-IP, label-free quantitative nano LC-MS/MS, and quantitative *in situ* PLA as an integrative methodological approach/workflow for conducting proteome-wide analysis of *H. sapiens* CLR interactome in primary human cells. Using this workflow, we identified and quantified 37 novel interactors of CLR in the context of the cellular proteome, which consists of 4902 members. Altogether, our new approach, generated resources (two datasets) and findings present a robust and valuable platform for advancing the discovery and comprehensive/proteome-wide characterization of physiologically relevant CLR interactome in a range of cell types in future studies, and thus for enabling the progress of both fundamental and preclinical research in the field.

### HDLEC proteome is at least eight times greater than previously known

The proteome coverage of 4902 proteins in HDLEC in our dataset was unexpected since it was almost 9-fold higher than an analogous study in which data-dependent acquisition mode was also used to identify 561 proteins (62). Such a large-scale proteome dataset could be exploited as a rich and valuable resource for future studies investigating EC (lymphatic and blood vessel) biology. Importantly, HDLEC proteome profiling revealed the landscape of transmembrane receptors and other signaling molecules. From 34 identified transmembrane receptors, 27 have not been previously reported in *in vitro* cultured HDLEC (62). These include ten GPCRs, three of which are known to play a role in lymphatics (63, 64, 65), while the roles for other seven (belonging to families of adhesion GPCRs and somatostatin receptors) remain to be investigated. The expression of these GPCRs in HDLEC suggests that they may have important, previously unappreciated, functions in the biology of these cells, alongside CLR.

### Novel label-free quantitative interaction proteomics platform for studying physiologically relevant CLR

Our novel integrative methodological approach provides a conceptual advance over former studies in which nonendogenous models were predominantly used along with the identification of CLR interactions with other proteins in a not “native” state. This is because it helped to overcome technological challenges in the field of GPCR research and generated a robust platform for dissecting physiologically relevant CLR interactome at a proteome-wide level not only in HDLEC but also in other primary human cells (including neurons, cardiomyocytes, vascular smooth muscle, and cancer/malignant cells) and tissues, where this GPCR plays important roles (13, 17, 22, 25). The characterization of such interactions will yield new biological insights about the mechanisms regulating the properties and function of CLR in physiological and pathological conditions.

### Previously unrecognized compositional complexity of the CLR interactome

In our study, we identified the interactions of endogenously expressed CLR with 37 novel proteins, which are associated with various cellular compartments and belong to specific classes.

Previous reports using animal tissues or tagged receptor overexpression in mammalian immortalized cell line models demonstrated that CLR interacts with RAMP 1, 2, and 3 and receptor component protein but without further investigation of other interactors (32, 36, 41). In our study, primary HDLEC expressed CLR (both core-glycosylated and terminally glycosylated forms) at endogenous levels, with localization in perinuclear space and at the cell surface, but RAMPs were not identified by LC-MS/MS in CLR IP samples or cellular proteome. This could be due to low abundance of endogenously expressed RAMPs in HDLEC, limitations in their detection by LC-MS/MS or differences in experimental conditions (*e.g.*, prestimulation *versus* poststimulation with agonists) and methodology used in overexpression models (*e.g.*, tagging, cross-linking *etc*.), when compared to our novel approach/methodology designed for and focused on studying physiologically relevant CLR interactome. Interestingly, the subcellular compartments in which members of identified by us CLR interactome exert their roles include ER, intracellular vesicles, and cell membrane (Fig. 6*B* and Table S5). These findings are in agreement with reports on subcellular localization of core-glycosylated and terminally glycosylated forms of unstimulated CLR, both upon tagging and overexpression in immortalized cells (11, 32, 35, 36) or endogenous expression in primary human EC cultured *in vitro* (11, 66).

Furthermore, our data reveal that CLR interacts with specific kinases, transporters, transcriptional, and translational modulators. The discovery (identification and quantification) of such distinct interactome uncovers its previously unrecognized compositional complexity and suggests a potential contribution to mechanisms implicated in CLR function. More specifically, our findings about direct interactions of CLR with kinases (CaMK2D and MRCKB), proteins related to RNA metabolism (IRP1), protein quality control, and trafficking regulators (VCP and Nup93, respectively) advance a fundamental understanding of the biology of this GPCR.

### Association of CLR interactome with GPCR signaling, posttranslational modification, and trafficking

CLR direct interactors CaMK2D, Nup93, and VCP have been previously shown to interact with and affect the signaling, posttranslational modifications, and trafficking of other GPCRs in different cell types (67, 68, 69). In particular, CaMK2 (calcium/calmodulin-dependent protein kinase 2) mediates phosphorylation of focal adhesion kinase upon agonist-activation of several GPCRs (*e.g.*, bombesin, vasopressin, or bradykinin) (67). VCP is involved in the polyubiquitination of β2-AR in ER membranes and Nup93 is required for a proper export to the plasma membrane and ligand-induced internalization, while also affecting β2-AR signaling (68, 69). Since these members of CLR interactome have the capacity to affect the function of other GPCRs, it is likely that CLR properties and signaling in HDLEC may also be regulated by them in a similar fashion.

### Functions of CLR interactome members in ECs and the lymphatic system

To our knowledge, the roles in HDLEC, lymphatic system biology and pathophysiology for any of the identified in our study five proteins which directly interact with CLR are currently unknown. However, the functions of CaMK2D and MRCKB in other EC types have been reported (70, 71, 72). CaMK2D/*CAMK2D* knockdown in human umbilical vein EC and human retinal microvascular EC reduced thrombin-induced permeability/barrier dysfunction through activation of Rho kinase (ROCK) A or growth factors–induced migration and proliferation, respectively (71, 72). MRCKB/*CDC42BPB* knockdown in human umbilical vein EC inhibited forskolin-induced stabilization of cell–cell junctions (70). Furthermore, the roles of some other, identified in our study, CLR interactome members in the lymphatic system have been described. In particular, lymphatic-specific deletion of Ras-interacting protein 1 (an endothelial-specific regulator of GTPases), Ras-related protein 1 (encoding for Ras-like small GTPase Ras-related protein 1), *Cdc24* or *Calclr* led to dilated lymphatics, edema, and disorganized cell junctions (25, 73, 74, 75, 76), Ras-related protein 1 *A/B* knockdown impairs AM-induced junctional tightening in neonatal HDLEC (74). Interestingly, CDC42 is regulated by Ras-interacting protein 1 (73) and promotes actin organization and cell migration through kinases MRCKA and MRCKB (77), with all three proteins being identified as members of the CLR interactome. In the context of these studies, our findings suggest that novel CLR interactors identified in our study are associated with reported roles for this GPCR and its agonists in proliferation, migration, and barrier function maintenance/monolayer stability of lymphatic endothelium *in vitro* and in lymphatic system development and/or function (13, 78, 79, 80). This warrants further investigation to determine whether targeting the CLR interactome in human cells would affect the properties and function of this GPCR and produce phenotype(s) which are similar to those currently associated with reduced expression of CLR/*CALCRL* in lymphatic and other systems.

### Direct interactors of CLR play functional roles in a range of cell types and diseases where this GPCR is implicated

The involvement of identified in our study CLR interactors in regulating functions of other cell types and their roles in several pathologies have been reported (81, 82, 83, 84, 85, 86, 87, 88, 89, 90, 91, 92, 93, 94). CLR is expressed in VSMC and cardiomyocytes, while its three agonists influence vasodilation, vascular permeability, and cardiac function, and are implicated in the pathophysiology of hypertension, atherosclerosis, ischemia, cardiac hypertrophy, and heart failure (19, 21, 22, 95). CaMK2D regulates proliferation and migration in VSMC, and apoptosis in cardiomyocytes, while CAMK isoforms are involved in the physiology and pathophysiology of the cardiovascular system (81, 82, 83, 84, 85, 86, 87). The knockdown of *nup93* promotes apoptosis in cardiomyocytes and its aberrant upregulated or downregulated expression is associated with dilated cardiomyopathy and coronary heart disease, respectively, in mice (88, 89). CLR is also expressed in neurons of human trigeminal ganglia and its agonist CGRP is involved in migraine pathogenesis and cluster headache (96, 97). CaMK2D is required for the induction of nerve injury-induced tactile allodynia in mouse hypothalamic and rat dorsal root ganglion neurons, respectively (90, 91).

Furthermore, upregulation of CLR and AM expression in cancer cells and tissues is associated with higher tumor grade and shorter survival rates in clear cell renal cell carcinoma patients (17). High expression of Nup93 in clear cell renal cell carcinoma tissue is associated with reduced overall survival of the patients (98). CLR expression is upregulated in acute myeloid leukemia bone marrow biopsies and cancer cell lines, where it impedes colony formation and is associated with an undifferentiated stage which is linked to poor prognosis and resistance to therapy (15, 23). In acute myeloid leukemia, inhibition of VCP promotes apoptosis *via* increased ubiquitination of proteasome components, autophagy-related proteins, and DNA damage response factors, impairs colony formation in malignant cells *in vitro*, while decreases disease load and prolonged survival of mice (93, 94). Collectively, these reports demonstrate that CLR and its direct interaction partners can be coexpressed and play important roles in a range of cells and conditions (physiological and pathological), where this GPCR and its agonists are implicated (21, 95, 99).

### Concluding remarks

In summary, the application of our novel integrative methodological approach/workflow generated a comprehensive proteome of HDLEC and *H. sapiens* CLR interactome datasets. Our first-of-its-kind study identified new signaling components of HDLEC proteome and revealed previously unrecognized complexity of CLR interactome, thus advancing the fundamental understanding of the biology of this GPCR. Collectively, our novel approach, resources, and findings form a unique platform which will serve as a springboard for facilitating rapid and comprehensive characterization of physiologically relevant CLR interactome at a proteome-wide level in a range of human cells and tissues, where this GPCR plays a role in health and disease.

## Experimental procedures

### Primary HDLEC

HDLEC from a 29-year-old female donor were obtained from PromoCell (Cat# C-12217). Cells have been tested by the manufacturer for the absence of human immunedeficiency virus (HIV-1 and HIV-2), hepatitis B virus (HBV), hepatitis C virus (HCV), human T-lymphotropic virus (HTLV-1 and HTLV-2), and microbial contaminants and double-checked by us for the lack of *mycoplasma* using EZ-PCR *Mycoplasma* Test kit (Biological Industries; Cat# 20-700-20) and the HyperLadderTM 1 kb (Bioline/Meridian Bioscience; Cat# BIO-33026). HDLEC were cultured as previously described (13). Briefly, cells were seeded onto a T-75 precoated flask and supplemented with PromoCell EC growth medium MV2 (Cat# C-22121) with the addition of recombinant human vascular endothelial growth factor C (VEGF-C) (R&D Systems; Cat# 9199-VC; 7.5 ng/ml). Cultures were incubated at 37 °C in a 5% CO2 humidified atmosphere and the medium was replaced every 24 h. Cells were passaged 1:2 at confluence (∼80%) by release with trypsin/EDTA. HDLEC were characterized by IF.

### Antibodies

Primary and secondary antibodies were obtained from a range of manufacturers and used at dilutions and concentrations described below. Immunoglobulin G (IgG) isotype controls were used at matched concentrations. Rabbit polyclonal anti-human CLR antibody in the form of serum (LN-1436; dilution 1:1000) was raised and characterized by us using pre-immune serum as control (dilution 1:1000) (11). Primary mouse monoclonal MRCKA (Cat# sc-374568, RRID: AB_10987859); MRCKB (Cat# sc-374597, RRID: AB_10988949), IRP1 (*ACO1*) (#sc-166022), nucleoporin 93 (Cat# sc-374400, RRID: AB_10988261), ERGIC-53 (*LMAN1*) (Cat# sc-365158, RRID: AB_10709004), CaMKD (Cat# sc-100362, RRID: AB_2068097), glycogenin 1 (Cat# sc-271109, RRID: AB_10610491), Bag-2 (Cat# sc-101216, RRID: AB_2062589), and XTP3TPA (*DCTPP1*) (Cat# sc-398501) all from Santa Cruz Biotechnology and used at 3.0 μg/ml. Mouse monoclonal p97 (*VCP*) (Cat# 612182, RRID: AB_399553; 3.0 μg/ml), calnexin (Cat# 610523, RRID: AB_397883; 3.0 μg/ml), cluster of differentiation 31 (or PECAM-1) (Cat# 555444, RRID: AB_395837; 5.0 μg/ml), mouse IgG1 (Cat# 555746, RRID: AB_396088), and mouse IgG2 (Cat# 555740, RRID: AB_396083) kappa isotype controls were all from BD Biosciences. Goat polyclonal prospero homeobox protein 1 (Cat# AF2727, RRID: AB_2170716; 2.0 μg/ml), LYVE-1 (#AF289; 2.0 μg/ml), and goat IgG (Cat# AB-108-C, RRID: AB_354267) were from R&D Systems, anti-rabbit IgG light chain (Cat# NBP2-75935; dilution 1:10,000) was from Novus Biologicals. Secondary conjugated polyclonal donkey anti-rabbit IgG Alexa Fluor 488 (Cat# A-21206, RRID: AB_2535792; 3.3 μg/ml), anti-rabbit IgG Alexa Fluor 594 (Cat# A-21207, RRID: AB_141637), anti-mouse IgG Alexa Fluor 488 (Cat# A-21202, RRID: AB_141607, 3.3 μg/ml), anti-goat IgG Alexa Fluor 488 (Cat# A-11055, RRID: AB_2534102, 3.3 μg/ml), and anti-goat IgG Alexa Fluor 594 (Cat# A-11058, RRID: AB_2534105, 3.3 μg/ml) all from Invitrogen, goat horseradish peroxidase (HRP) anti-mouse IgG (Cat# P0447, RRID: AB_2617137; dilution 1:1000), and anti-rabbit IgG (Cat# P0448, RRID: AB_2617138; dilution 1:1000) were from Dako.

### Immunofluorescence

IF was used for HDLEC characterization/authentication/phenotyping and antibody testing, utilizing the previously described method (11). Briefly, HDLECs were subseeded in 8-well slide chambers (5000 cells per well) and allowed to reach 80% confluency. Cultured cells were washed once with phosphate-buffered saline (PBS; Thermo Fisher Scientific, Cat# 10209252) and fixed using either 4% paraformaldehyde (PFA) or acetone/methanol (2:3 ratio) solutions. After aspirating the PFA solution, cells were washed once in PBS and stored in PBS (pH 7.2) at 4 °C until required. After acetone/methanol fixation, cells were left air-dry for 25 min and then stored at −20 °C until required. A preblocking step, using 10% donkey serum, which was diluted in PBS containing 0.1% Triton X-100 “dilution buffer” was performed for 30 min at room temperature (RT) prior to incubation with primary antibody. Primary antibodies were diluted at appropriate concentrations in 2% donkey serum in dilution buffer, added to cells and incubated overnight at 4 °C. Incubation of human CLR immune serum with 10 μg/ml of the immunizing peptide was used as a negative control for immunostaining (11, 17). Cells were washed three times with PBS before the incubation with appropriate secondary antibody diluted in 2% donkey serum in dilution buffer. Incubation with appropriate secondary fluorophore-conjugated antibodies was performed under light protection at RT for 45 min. Next, the secondary antibody solution was removed and the wells were washed three times. After PLA, incubation with Alexa Fluor 635-labeled phalloidin (Invitrogen, Cat# A34054, 1:100) for 40 min at RT in the dark. Mounting was done using 4′,6-diamidino-2-phenylindole (DAPI; VECTASHIELD Vibrance Antifade Mounting Medium with DAPI, Vector Laboratories, Cat# H-1800) and imaging (see section Microscopy and image analysis).

### Cell lysis and determination of protein concentration

Cell lysis was performed as previously described (11). Briefly, all steps were performed on ice. Cells were washed with ice-cold filtered PBS and homogenized using cell scrapers in radioimmunoprecipitation assay lysis buffer solution, in which protease (Thermo Fisher Scientific, Cat# A32965) and phosphatase inhibitor (Roche, Cat# 4906845001) cocktails were added. Samples were lysed by aspirating up and down and repeating three times at 10-min intervals. Insoluble material was pelleted at 13,000*g* for 10 min at 4 °C, and supernatants were stored at −20 °C. Bicinchoninic acid assay (Thermo Fisher Scientific, Cat# 23227) was used according to the manufacturer's instructions to determine total protein concentration in cell lysates. The measurements of absorbance at a wavelength of 562.0 nm were taken using a Tecan Infinite M200 Plate Reader (Cat# 30213615). Total cell lysates were processed for immunoprecipitation or immunoblotting or protein digestion and subsequent label-free quantitative nano LC-MS/MS analysis.

### Immunoprecipitation

A precleaning step was performed before IP. In brief, 2.0 μg of rabbit preimmune serum (11) per 1.2 mg (2.0 μg/μl) of total protein were incubated together with 4.0 mg of protein G magnetic beads (Invitrogen, Cat# 10007D) by head-over-tail rotation for 30 min at 4 °C. Next, beads were collected using a magnet and clear supernatants were transferred into fresh tubes. For IP, equal amounts of protein (600 μg; 2.0 μg/μl) were mixed with 2.7 μg of either rabbit anti-human CLR/immune serum LN-1436 (11) or preimmune serum (control) and incubated by head-over-tail rotation for 90 min at 4 °C. Both anti-human CLR/immune serum and preimmune serum were obtained from the same rabbit (11). Next, formed immune complexes were coupled to 4.2 mg of protein G magnetic beads and incubated by head-over-tail rotation for 60 min at 4 °C. The beads were washed three times using a buffer containing no detergent (50 mM Tris, pH 7.4, and 150 mM NaCl, protease and phosphatase inhibitors mentioned above) prior to subsequent elution steps. Immunoprecipitated samples were eluted by incubation at 55 °C under reducing conditions for 25 min before sodium dodecyl-sulfate polyacrylamide gel electrophoresis (SDS-PAGE) and immunoblotting analysis. Alternatively, the washing buffer was removed and beads were stored at −80 °C before immune complexes were processed for on-bead protein digestion and label-free quantitative nano LC-MS/MS analysis.

### SDS-PAGE and immunoblotting

Protein lysates (total or after IP) were subjected to SDS-PAGE and immunoblotting as previously described (11). In brief, samples were electrophoretically separated on 10% polyacrylamide-based gel (acrylamide/methylene bisacrylamide solution at 37.5:1 ratio, 375 mM Tris pH 8.8, 0.1% SDS, 0.1% ammonium persulfate or APS (ammonium persulfate), and 0.04% tetramethylethylenediamine) set with 5% stacking gel (acrylamide/methylene bisacrylamide solution at 37.5:1 ratio, 126 mM Tris pH 6.8, 0.1% SDS, 0.1% APS, and 0.01% tetramethylethylenediamine). Electrophoresis was performed using Tris running buffer (25 mM Tris base, 0.192 M glycine, 0.1% SDS, pH 8.3) at 100 V for 2 h or longer until optimal resolution of proteins at 4 °C was achieved. Transfer to polyvinylidene difluoride membrane was performed using Tris-based transfer buffer (25 mM Tris base, 0.192 M glycine, pH 8.3) at 60 V for 3 h at 4 °C. The membranes were incubated in a blocking solution (5% nonfat milk in 20 mM Tris-buffered saline pH = 7.4 containing 0.5% Tween-20 (TBS/T buffer) for 60 min prior to incubation with primary antibody overnight in a blocking solution on a tube roller at 4 °C and subsequent incubation (45 min) with secondary HRP-conjugated antibody. Next, membranes were washed three times at 5-min intervals using TBS/T buffer and HRP activity was detected using an enhanced chemiluminescence kit (Bio-Rad, Cat# 1705061). After detection, the membranes were stripped using stripping buffer (Thermo Fisher Scientific, Cat# 10016433), re-probed, or stored at −20 °C. Prestained molecular weight markers (Abcam, Cat# ab116028) were used to estimate the molecular weight of the bands. Enhanced chemiluminescence for the detection of HRP-conjugated antibodies was used. Imaging and densitometry were performed using Bio-Rad ChemiDoc XRS+ and Bio-Rad Image Lab 6.0 software (https://www.bio-rad.com/en-uk/product/image-lab-software?ID=KRE6P5E8Z, RRID:SCR_014210) as previously described (100). Briefly, rolling desk background subtraction from total protein lane density with a disk size of 5.0 mm was applied to all lanes. Relative quantification for each band was performed upon comparison to the reference band (rabbit IgG light chain in control IP). Exposure times relied on the quality and intensity of the obtained signal.

### Statistical analysis of immunoblotting data

IP and immunoblotting experiments were performed in quadruplicates. Densitometry data was normal as analyzed using D'Agostino-Pearson showing data normality, followed by two-tailed unpaired Student’s *t* test for both core-glycosylated and terminally glycosylated forms of CLR. GraphPad Prism 8 software (https://www.graphpad.com/) was used for the statistical analysis. Specific statistical tests used for individual experiments are specified in individual Figure legends.

### Protein digestion and peptide clean-up

Immune complexes and total cell lysates were subjected to proteolytic digestion, desalting, and label-free quantitative nano-LC-MS/MS. For on-bead-digestion of immunoprecipitated samples, the enzyme slurry was resuspended with 4 M urea in 20 mM Hepes (pH 8.0) solution. Immune complexes were incubated with 1.5 μg of LysC/trypsin solution (Promega, Cat# V5071, concentration 1.0 μg/μl) for 6 h at 37 °C. LysC is active at 4 M urea. Next, the bead slurry was diluted using Hepes and dithiothreitol (DTT) (2.0 mM) solution to reduce the urea concentration to 1.0 M and DTT to 1.0 mM, respectively, and activate the trypsin. Samples were then incubated overnight at 37 °C. For alkylation and desalting, iodoacetamide (5 mg/ml) was added to the samples and then incubated for 30 min in the dark. Samples were treated with 1 μl trifluoroacetic acid (TFA) to stop the digestion and desalted in C18 stage tips. For single-pot solid-phase-enhanced sample preparation magnetic bead digestion of total cell lysates, a single-pot, solid-phase-enhanced sample preparation method was used (101). Protein samples were mixed with reconstitution buffer (50 mM Hepes, pH 8, 1% (wt/vol) SDS, 1% (vol/vol) Triton X-100, 1% (vol/vol) NP-40, 1% (vol/vol) Tween 20, 1% (wt/vol) deoxycholate, 5 mM ethylenediaminetetraacetic acid (EDTA), 50 mM NaCl, 1% (vol/vol) glycerol). Reducing agent stock (500 mM of DTT) was added to a final concentration of 5 mM DTT. Next, samples were heated using a Thermomixer at 60 °C for 30 min, mixing at 1000 rpm. Alkylating agent (chloroacetamide) was added to a final concentration of 20 mM and the reaction was allowed to proceed for 30 min at RT. A total of 100 μg of magnetic beads stock solution (50 μg/μl) were added to 10 μg of reduced and alkylated protein sample. A 100% ethanol solution was added (to achieve a final concentration of approx. 60%), and the solution was homogenized. The binding mixture was incubated in a Thermomixer at 25 °C for 5 min at 1000 rpm. The unbound supernatant was removed using a magnet and the beads were re-suspended in 80% ethanol solution. Next, the rinse was removed and 100 μl of digestion solution (100 mM ammonium bicarbonate, pH 8.0 in water) containing 0.4 μg of trypsin per tube (for 1:50 trypsin to protein ratio) was added. Samples were sonicated for 1 min on an ultrasonic water bath and the fully reconstituted beads were incubated for 18 h at 37 °C in a Thermomixer at 1000 rpm. Digested samples were centrifuged at 20,000*g* for 1 min, and the supernatant was transferred into a fresh tube for each sample. Peptides were dried in a speed-vac and resuspended in 0.1% TFA prior to MS analysis.

### Label-free quantitative nano LC-MS/MS

For MS data acquisition, peptides were analyzed on a Q Exactive Plus Hybrid Quadrupole-Orbitrap mass spectrometer (Thermo Fisher Scientific, Cat# IQLAAEGAAPFALGMBDK) connected to an UltiMate 3000 Rapid Separation Liquid Chromatography (RSLC) system (Thermo Fisher Scientific, Cat# ULTIM3000RSLCNANO. A total of 5 μl of tryptic peptides was loaded for each sample onto a homemade column in (Medical Research Council Laboratory of Molecular Biology; 100 mm length, 75 μm inside diameter [i.d.]) packed with 1.9 μm ReprosilAQ C18 (Dr Maisch, Cat# r119.aq). Peptides were separated by reversed-phase chromatography using an increasing acetonitrile gradient (3–32%) over 40 min and at a flow rate of 250 nl/min. The mass spectrometer was operated in a positive ion mode with a capillary temperature of 220 °C, with a potential of 2000 V applied to the column. Data were acquired with the mass spectrometer operating in automatic data-dependent switching mode, selecting the 12 most intense ions prior to MS/MS analysis. MS Proteomics data have been deposited at the ProteomeXchange Consortium *via* the PRIDE partner repository (102) with the dataset identifier PXD032156.

### MS data processing with MaxQuant

MS data processing and analysis were conducted as previously described (103). Analysis of the raw data was performed using the MaxQuant Linux version (Max-Plank Institute of Biochemistry, https://maxquant.org/, RRID:SCR_014485) with the built-in Andromeda search engine (104, 105) on VIPER High-Performance Computing hardware (University of Hull). LFQ normalization was performed by analyzing raw LC-MS/MS data (raw peptide intensities) using MaxQuant and enabling the MaxLFQ algorithm (106). Specific approaches and samples used for LFQ normalization are specified in individual Figure legends. The spectra were searched against the human UniProtKB/Swiss-Prot database version 06/2021 (canonical sequence). The MaxQuant default settings (including mass tolerance) were used. Specific settings: trypsin as the protease (two missed cleavages); carbamidomethylation (Cys) as the fixed modification; oxidation (Met), phosphorylation (Ser, Thr, Tyr), and N-terminal protein acetylation as variable modifications. The FDR was set to 1% for both peptide and protein levels, and the minimum peptide length was set to seven amino acids. Quantification was performed using the LFQ algorithm from MaxQuant (106). Only proteins identified with at least one peptide at FDR <1% were considered for further analysis.

### Statistical analysis of MS data

Mean LFQ intensities were calculated from technical duplicates for each of four independent experiments per condition in MaxQuant with peptide, and protein FDRs set to 1%. LFQ intensity values were transformed to log_2_ values and proteins quantified in fewer than 75% of all samples were excluded. Furthermore, data was cleared of reversed hits, contaminants (n = 99) and “only identified by site.” Missing values were imputed from a width-compressed, down-shifted normal distribution, using Perseus (Max-Plank Institute of Biochemistry, https://maxquant.net/perseus/, RRID:SCR_015753) version 1.6.7.0 (107). The mean copy numbers and mean abundance (in protein mass and molecules) of proteins per diploid nucleus were estimated using the “proteomic ruler” package in Perseus (53). Prior to statistical analysis of IP data, potential bead cytoplasmic and nuclear contaminants (as previously described (108)) were identified in both CLR and control IP samples (Table S2) and removed. For MS data, two-tailed unpaired Student’s *t* tests with a permutation-based on FDR-adjusted *p* value <0.0075 (FDR threshold of 1% applying 1000 randomizations) were conducted for multiple hypothesis testing correction. A fold-change cut-off of 3.5, which represents the mean plus two SDs of the distribution of fold change in log_2_ LFQ intensity across all proteins was used to determine high-confidence hits (correspond to proteins interacting with CLR), as previously described (109). D'Agostino-Pearson test was used to assess the normality of LFQ intensity data for IP samples and HDLEC total cell lysates prior to the analysis of CLR expression (*p* < 0.05; nonparametric data), and was followed by the Mann–Whitney test. Results were deemed significant if *p* < 0.05 and were denoted as: ∗*p* < 0.05 and ∗∗∗*p* < 0.001. GraphPad Prism 8 software was used for the statistical analysis. Specific statistical tests used for individual experiments are specified in individual Figure legends.

### *In situ* PLA

Prior to *in situ* PLA, cell fixation (1% PFA) and primary antibody incubation steps are the same as for IF. Primary mouse mAbs raised against proteins of interest were selected based on high specificity (according to information provided by manufacturers and additional literature search; data not shown) for investigating the expression of 11 out of the top 20 enriched in IP proteins. Upon their detectable expression in HDLEC, CaMK2D, NUP93, MRCKB, ERGIC-53, VCP, and IRP1 (Fig. S2) were further selected for the validation of label-free quantitative nano LC-MS/MS findings by quantitative *in situ* PLA analysis. Duolink *In Situ* PLA Probe Anti-Rabbit PLUS (Sigma-Aldrich, Cat# DUO92002-100RXN) and anti-mouse MINUS (Sigma-Aldrich, Cat# DUO92004-100RXN) along with Duolink *In Situ* Detection Reagents Red (Sigma-Aldrich, Cat# DUO92008-100RXN) were used, and PLA assay was performed according to manufacturer’s instructions (Sigma-Aldrich). Mounting was done using DAPI (VECTASHIELD Vibrance Antifade Mounting Medium, as described above (see section Immunofluorescence) and imaging as described below (see Microscopy and image analysis).

### Microscopy and image analysis

After IF and *in situ* PLA, the fixed cells were examined using an ZEISS enhanced contrast Plan-Neofluar 20x/0.5 Ph2 M27 objective and an LSM 710 confocal system with AXIO Observer Z1 microscope (Carl Zeiss Microscopy) and 405-, 488-, 561-, 633-nm laser lines. Images were acquired using ZEN Black edition SP7 FP3 (version 14.0; Carl Zeiss Microscopy) at RT. Image processing and analysis were performed using ImageJ/Fiji (110) (National Institutes of Health, https://imagej.net/software/fiji/, RRID: SCR_003070) and Zen Blue edition (version 3.0; Carl Zeiss Microscopy). All PLA images were obtained using z-stacks of 10 to 12 images of 0.9 μm between each focal plane and maximum projections were produced. Fiji/ImageJ was used for the semiautomatic quantitative assessment of PLA dots (111). In brief, based on the Alexa Fluor 635 phalloidin staining, an F-actin mask (gaussian blur filter, subtract background, auto threshold “method=default”) was created to measure the size of each cell based on actin cytoskeleton staining. The mask was used to count PLA signals (dots) using the Fiji/ImageJ option “find maxima” and possible off-target (outside the cytoskeleton) signals for each cell were excluded.

### Statistical analysis of *in situ* PLA data

Statistical analysis of EC area (μm^2^) and *in situ* PLA signal (dots per cell) was based on the quantification of 20 cells per condition. Shapiro–Wilk test was used for analyzing data normality. In EC area (μm^2^) analysis, datasets that had normal distribution were ERGIC-53 and VCP and an unpaired Student *t* test was used for statistical analysis. Kruskal–Wallis test was used for the comparison of EC areas for the rest of the proteins. In the *in situ* PLA signal dataset, IgG1, IgG2, and MRCKB datasets passed the normality test and an unpaired Student’s *t* test was used for the IgG2 *versus* MRCKB comparison, while the Mann–Whitney test for the rest. GraphPad Prism 8 software was used for the statistical analysis. Specific statistical tests used for individual experiments are specified in individual figure legends.

### Extraction of CLR peptide data

CLR peptide data was extracted from the ProteomeXchange dataset PXD019909 obtained from primary ECs isolated from human skin (54) and used for comparative analysis against our dataset (Table 3).

### GO analysis

Functional profiling of the proteomic data was performed using the GO resource from the GO Consortium server (112). Functional enrichment analysis of overrepresented ontology terms was performed with the GO Enrichment Analysis tool powered by PANTHER (113, 114). This enabled the categorization of molecular function, biological process, and cellular localization of the unique proteins (members of CLR interactome) identified in our study.

## Data availability

The data underlying Figure 1, Figure 2, Figure 3, Figure 4, Figure 5, Figure 6, Figs. S1–S3, Table 1, Table 2, Table 3, and Tables S1–S3 are available in the main paper and its supplemental material. The data underlying Figure 2, Figure 3, Figure 4 and 6, Table 1, Table 2, Table 3, and Tables S1–S3 are openly available in the ProteomeXchange Consortium *via* the PRIDE partner repository with the dataset identifier PXD032156. The data underlying Table 3 are openly available in the ProteomeXchange Consortium *via* the PRIDE partner repository with the dataset identifier PXD019909. Any additional information required to reanalyse the data reported in this paper is available from the lead contact upon reasonable request.

## Supporting information

This article contains supporting information (11, 108).

## Conflict of interest

The authors declare that they have no conflicts of interest related to the contents of this article.

## Figures and Tables

**Figure S3:**