# Supplementary material for: Quantitative proteomics reveals CLR interactome in primary human cells
Source: J Biol Chem. 2024 May 20;300(6):107399. doi: 10.1016/j.jbc.2024.107399 (PMC11231609; doi:10.1016/j.jbc.2024.107399)
Supplement: Supporting Information [file mmc1.docx]

**Supporting figure legends**

**Supporting Figure S1. Quantification of fold-change difference in protein abundance between cellular proteome and CLR IP samples in human dermal lymphatic endothelial cells**. Box and whiskers plot showing the protein abundance in the total cell lysate/cellular proteome of human dermal lymphatic endothelial cells (HDLEC) and upon calcitonin receptor-like receptor (CLR) immunoprecipitation (IP). Protein abundance was assigned by label-free quantitation (LFQ) intensity acquired for all proteins upon liquid chromatography-tandem mass spectrometry. The data represents normalized (between IP and total cell lysate/cellular proteome samples) log_2_ LFQ values for all proteins identified in the Total (n=4,902) and IP (n=642) groups. Box and whiskers plot represents median values (n=4 independent biological replicates). The box contains the 25th and 75th percentiles and whiskers are the minimum and maximum mean LFQ values of each dataset. Statistical analysis was performed using the D'Agostino-Pearson test (p<0.001) followed by the Mann-Whitney test (ns: not significant).

**Supporting Figure S2. Expression of 11 selected novel CLR interactors in human dermal lymphatic endothelial cells analyzed by immunofluorescence**. Human dermal lymphatic endothelial cells (HDLEC) were cultured *in vitro* and fixed in paraformaldehyde (see Experimental procedures for full experimental details). **A and B**, isotype controls IgG1 and IgG2 (top panels) were used at matched concentrations to primary mouse monoclonal antibodies to analyse the expression of 11 selected proteins identified by label-free mass spectrometry as CLR interactors (encoded by *LMAN1, VCP, CANX, BAG2, GYG1, CDC42BPA, CDC42BPB, ACO1, NUP93, CAMK2D* and *DCTPP1* genes). Secondary Alexa Fluor 488-conjugated donkey anti-mouse antibody (white pseudocolor; white arrows) was used to detect the signal for each protein. Nuclei were counterstained using DAPI (blue). Scale bars represent 20 μm.

**Supporting Figure S3. Detection of endothelial cell area for *in situ* proximity ligation assay.** Human dermal lymphatic endothelial cells (HDLEC) were cultured *in vitro* and fixed in paraformaldehyde. See Experimental procedures for full experimental details. Expression of F-actin based on Alexa Fluor 635 phalloidin staining (white pseudocolor) was used for quantification analysis of endothelial cell area in μm^2^ for *in situ* proximity ligation assay (see **Fig. 5B**; see Experimental procedures). Image analysis was performed using F-actin (cytoskeleton) staining as a mask to designate the perimeter of each cell in six protein and relevant isotype control groups (top) IgG2, CaMK2D, Nup93, IRP1 and (bottom) IgG1, MRCKB, VCP and ERGIC-53. Cell area was used for *in situ* PLA signal quantification (see **Fig. 5B**). Nuclei were counterstained with DAPI (blue). Scale bars represent 10 μm.

**Supporting Figure S4. Detection of CLR and Nup93 in IP samples**. Immunoblotting analysis of human dermal lymphatic endothelial cell (HDLEC) total cell lysates/cellular proteome (Input) and samples acquired from immunoprecipitation (IP) using anti-human CLR antibody/immune serum (anti-CLR) and pre-immune serum (Control). See Experimental procedures for further details. Membranes were sequentially probed for calcitonin receptor-like receptor (CLR), and then stripped and reprobed for nucleoporin-93 (Nup93) (see Experimental procedures for further details). The molecular size markers are shown in kilodaltons (kDa).

**Supporting table legends**

**Supporting Table S1. Quantitative analysis of human dermal lymphatic endothelial cell proteome**. Identification and quantification of the proteome of *in vitro* cultured primary human dermal lymphatic endothelial cells (HDLEC) by using label-free quantitative nano liquid chromatography-tandem mass spectrometry (nano LC-MS/MS). 56,930 peptides and 5,102 protein groups were identified and 4,902 protein groups were quantified. Detailed information about label-free quantitation (LFQ) intensity, number of identified peptides, percentage of sequence coverage, peptide to spectra matches along with number of estimated copy numbers and number of molecules of specific proteins per total protein molecules are indicated. Four independent experiments were performed.

**Supporting Table S2. Potential bead contaminants identified in both CLR IP and control IPs**. List of potential cytoplasmic and nuclear contaminants associated with non-specific binding to the protein G magnetic beads (108) and identified in both CLR IP and control IP in our study. The gene and protein names for each potential contaminant are indicated.

**Supporting Table S3. Quantitative analysis of proteins co-immunoprecipitated with CLR endogenously expressed in human dermal lymphatic endothelial cells**. Identification and quantification of proteins co-immunoprecipitated (co-IP) with calcitonin receptor-like receptor (CLR) from primary human dermal lymphatic endothelial cell (HDLEC) was done by using label-free quantitative nano-liquid chromatography-tandem mass spectrometry (nano LC-MS/MS). Proteins interacting with CLR were those with absolute fold change in label-free quantitation (LFQ) intensity ≥ 3.5 (mean + 2 SD) and 1% FDR-adjusted p-value<0.0075 in Co-IP proteome captured by using anti-CLR antibody/immune serum LN-1436 (11) compared to pre-immune serum.

**Supporting Table S4. Abundance of proteins exclusively identified in CLR immunoprecipitation samples**. List is showing the mean log2 label-free quantitation (LFQ) intensity values of proteins identified only in CLR immunoprecipitation (IP) samples and not in control IP for all four independent experiments (the range is 20.30-29.17 A.U.). Proteins have been ordered from higher to lower according to their mean LFQ values and indicated by gene encoding them. CLR (encoded by CALCRL gene) is highlighted in red.

**Supporting Table S5. Protein class and sub-cellular localization analysis of CLR interactome**. ANalysis THrough Evolutionary Relationships (PANTHER) Protein Class and Gene Ontology (GO) Cellular Compartment (CC) analyses of 37 identified in this study members of calcitonin receptor-like receptor (CLR) interactome in human dermal lymphatic endothelial cells (HDLEC). Genes, proteins, protein classes and predicted cellular compartments are listed. Panther Class and complete CO CC identifiers are indicated. Predicted cellular compartments associated with each protein are marked with a cross.
